# Microbial Changes in the Periodontal Environment Due to Orthodontic Appliances: A Review

**DOI:** 10.7759/cureus.64396

**Published:** 2024-07-12

**Authors:** Mona A Al-Mutairi, Lamia Al-Salamah, Lubna A Nouri, Bandary S Al-Marshedy, Noura H Al-Harbi, Entesar A Al-Harabi, Hend A Al-Dosere, Farah S Tashkandi, Zainab M Al-Shabib, Abdulaziz M Altalhi

**Affiliations:** 1 Periodontics, Ministry of Health, Riyadh, SAU; 2 Endodontics, Ministry of Health, Riyadh, SAU; 3 Family Dental Medicine, Ministry of Health, Riyadh, SAU; 4 Dentistry, Ministry of Health, Riyadh, SAU; 5 Dentistry, Ministry of Health, Makkah, SAU; 6 Dentistry, Ministry of Health, Aljish, SAU

**Keywords:** clear aligners, oral microbiota, orthodontic care, periodontitis, perio-ortho, ortho-perio

## Abstract

Orthodontic appliances significantly influence the microbiological dynamics within the oral cavity, transforming symbiotic relationships into dysbiotic states that can lead to periodontal diseases. This review synthesizes current findings on how orthodontic treatments, particularly fixed and removable appliances, foster niches for bacterial accumulation and complicate oral hygiene maintenance. Advanced culture-independent methods were employed to identify shifts toward anaerobic and pathogenic bacteria, with fixed appliances showing a more pronounced impact compared to clear aligners. The study underscores the importance of meticulous oral hygiene practices and routine dental monitoring to manage these microbial shifts effectively. By highlighting the relationship between appliance type, surface characteristics, treatment duration, and microbial changes, this review aims to enhance dental professionals' understanding of periodontal risks associated with orthodontic appliances and strategies to mitigate these risks. The findings are intended to guide clinicians in optimizing orthodontic care to prevent plaque-associated diseases, ensuring better periodontal health outcomes for patients undergoing orthodontic treatment.

## Introduction and background

The oral cavity harbors the second richest microbial community in the human body, comprising over 700 species of bacteria that colonize both the hard tissues and soft mucosa [1,2]. These bacteria form a harmonious community driven by the host's defense system, thriving in three distinct areas: group 1, subgingival and supragingival; group 2, saliva, tongue, and hard palate; and group 3, cheek and sublingual area [2].

The balance between the host and the oral microbiome is crucial for both oral and overall health. Disruptions in this balance increase the risk of developing various oral diseases [3]. The terms "oral microflora," "oral microbiota," or "oral microbiome" refer to the microorganisms present in the human oral cavity [4]. Joshua Lederberg coined the term "microbiome" to denote the ecological community of commensal, symbiotic, and pathogenic microorganisms that inhabit our body space, historically overlooked as determinants of health and disease [5]. As noted, shifts in this microbial community can lead to major dental and periodontal issues, such as caries (white spot lesions), gingivitis, and periodontitis [6].

Orthodontic intervention, particularly with fixed appliances, is a notable factor that disrupts the equilibrium between the host and the oral microbiome. This procedure, increasingly popular among adolescents and adults due to technological advancements, often extends over one or more years and may negatively impact oral health [7]. Davis et al. [8] described how dental and periodontal complications can arise from direct gingival irritation due to excessive force exerted by appliances on the periodontium or from poor oral hygiene maintenance, leading to plaque accumulation, dental caries, and periodontal diseases [8]. Patients undergoing orthodontic treatment should be informed of the risks associated with instability and changes in plaque biofilm composition and should be taught preventive or control methods to ensure the successful completion of their therapy [6].

This narrative review aims to comprehensively assess the impact of orthodontic therapy on periodontal microbiology and health. The study explores key factors associated with microbial shifts, such as bacteria and fungi, the type of appliance used, and the treatment duration. The findings emphasize the need for controlling methodologies to mitigate the effects of orthodontic therapy on the oral microbiome and maximize treatment outcomes. However, it is important to note that the reviewed studies did not explore the impact of orthodontic therapy on the general/systemic health of the patients, highlighting the need for further research into the presence and effects of other microbial entities within the biofilm.

## Review

The oral cavity is home to the second richest microbial community in the human body, with over 700 species of bacteria colonizing both hard tissues (such as teeth) and soft tissues (such as the mucosa) [1,2]. These bacteria coexist harmoniously within three distinct areas: (1) subgingival and supragingival regions (around and above the gum line), (2) saliva, tongue, and hard palate, and (3) the cheek and sublingual (under the tongue) area [2].

Maintaining a balance between the host (the human body) and the oral microbiome (the community of microorganisms in the mouth) is essential for both oral and overall health. Disruptions in this balance can increase the risk of various oral diseases [3]. Terms such as "oral microflora," "oral microbiota," and "oral microbiome" all refer to the microorganisms present in the human mouth [4]. The term "microbiome" was coined by Joshua Lederberg to describe the ecological community of commensal (beneficial), symbiotic (mutually beneficial), and pathogenic (disease-causing) microorganisms that inhabit our bodies and significantly influence our health and disease states [5]. Changes in this microbial community can lead to significant dental and periodontal issues, such as cavities (white spot lesions), gingivitis (gum inflammation), and periodontitis (severe gum disease) [6-69].

Orthodontic treatment, especially with fixed appliances (braces), is a notable factor that can disrupt the balance between the host and the oral microbiome. This type of treatment, which has become increasingly popular among both adolescents and adults due to technological advancements, often lasts for a year or more and can negatively impact oral health [7]. According to Davis et al. [8], dental and periodontal complications can arise from direct gingival irritation caused by the excessive force of the appliances on the gums or from poor oral hygiene maintenance, leading to plaque buildup, cavities, and periodontal diseases. It is essential for patients undergoing orthodontic treatment to be aware of the risks associated with these changes in plaque biofilm composition and learn preventive measures to ensure the successful completion of their therapy [6].

This narrative review aims to provide a comprehensive assessment of the impact of orthodontic therapy on periodontal microbiology and health. The study examines key factors associated with microbial shifts, such as bacteria and fungi, the type of orthodontic appliance used, and the duration of treatment. The findings emphasize the importance of implementing control methodologies to mitigate the effects of orthodontic therapy on the oral microbiome and optimize treatment outcomes. However, it is important to note that the reviewed studies did not explore the impact of orthodontic therapy on the general/systemic health of patients, highlighting the need for further research into the presence and effects of other microbial entities within the biofilm.

Background

Unaltered Periodontal Microbiology

The human mouth hosts a diverse array of microbiomes, including bacteria, viruses, protozoa, fungi, and archaea. Researchers have identified thousands of bacterial species across various phyla, such as Actinobacteria, Bacteroidetes, Firmicutes, Proteobacteria, Spirochaetes, Synergistetes, and Tenericutes, and the uncultured divisions GN02, SR1, and TM7. It is noteworthy that approximately half of these uncultured bacteria remain undiscovered [9]. The oral microbiota, consisting of several thousand species, is an integral part of the oral cavity and plays a crucial role in protecting the host from extrinsic bacteria with disease potential, thereby impacting oral health [10,11].

Popova et al. [12] examined the relationships among bacteria in mature biofilms, discovering that these inter-bacterial relationships can influence the virulence of periodontal microflora either positively or negatively. Positive interactions, known as symbiosis, are categorized into mutualism, synergism, and commensalism. In mutualism, both species, such as *Porphyromonas gingivalis* with *Treponema denticola* and *Tannerella forsythia* with *Fusobacterium nucleatum*, benefit equally from their coexistence. Synergism occurs when the combined pathogenic potential of the interacting species exceeds the sum of their individual effects, as seen with *Porphyromonas gingivalis* and *Fusobacterium nucleatum*. Conversely, in commensalism, only one species benefits, such as the relationship between *Porphyromonas gingivalis* and *Campylobacter rectus*. Understanding these positive relationships within mature biofilms is essential, as they can either stimulate or inhibit the growth of specific bacterial species, thereby affecting the balance between the host and the microbiome [12].

Identifying Key Microbial Changes and Complications

Due to advancements in methodologies such as culture-independent, high-end technologies such as reverse transcriptase-polymerase chain reaction, it is now possible to identify the number of microbial species present in various samples, including saliva and supragingival and subgingival plaque [13]. The composition of dental plaque changes over time (from early to mature plaque) and varies depending on the location (supragingival or subgingival plaque groups). These variations are linked to different pathologies, such as caries and periodontitis [14].

As Wade et al. [9] described, dental caries and periodontal disease are the two most common diseases caused by bacteria. These diseases occur when a microbial shift transforms a symbiotic relationship into dysbiosis. Bacteria from the orange and red complexes, which are not grouped in the subgingival bacterial classification, are suspected of causing periodontal diseases [11]. Supragingival plaque primarily consists of cariogenic bacteria, such as Lactobacilli and Actinomycetes species, which lead to enamel demineralization [15]. Additionally, the role of supragingival microflora is crucial in periodontal pathogenesis; gram-negative anaerobes can co-aggregate and lead to periodontal diseases [16].

Subgingival microbiota/plaque has been the primary focus of periodontal pathogenesis research [12]. A detailed study by Socransky et al. [17] analyzed the different bacterial species found in subgingival plaque. These bacteria belonged to specific complexes, and their strong and weak associations with periodontal disease potential were evident. The bacteria in the green and yellow complexes were early colonizers that provided a foundation for more harmful bacteria from the orange complexes. The orange complex bacteria produced toxins, leading to clinical attachment loss and deepening pockets, and forming a base for the red complex bacteria to thrive. Thus, it was established that the ultimate damage to the periodontium was caused by red complex bacteria, and subgingival plaque formed a prime niche for these bacteria [17]. Several studies have explored the impact of orthodontic therapy on oral microflora. The general mechanism remains the same: orthodontic appliances complicate oral hygiene maintenance, leading to more plaque buildup and microbiological shifts, which can result in oral complications.

In a recent study, Chen et al. [18] aimed to provide the first longitudinal, culture-free, deep sequence profiling of subgingival bacteria in patients undergoing fixed appliance therapy at an early stage. Their study found an increased incidence of local gingivitis and mild periodontitis in subjects undergoing orthodontic treatment compared to an untreated control group. This was linked to a greater diversity of subgingival microbiomes and the discovery of 12 novel bacterial species [18].

Multiple studies have observed a shift in the supragingival microbiome, characterized by increased anaerobic bacteria, periodontal pathogens, and a reduction in commensal bacteria [19]. Under certain conditions, organisms that normally coexist peacefully can become detrimental. This is particularly true with orthodontic appliances, which might provide a breeding ground for opportunistic pathogens that require increased scrutiny [20]. One such commensal fungus, *Candida albicans*, naturally presents in the oral microflora of 53% of the general population and can become pathogenic due to an imbalance in the microbial colony following orthodontic treatment, combined with inadequate oral hygiene or a compromised host immune system, leading to candida-associated oral infections [21]. A few studies have reported an increase in caries-causing bacteria, including *Streptococcus mutans*, *Lactobacillus* spp., and potentially pathogenic gram-negative bacteria in patients with orthodontic appliances [13,22,23]. Another study confirmed the presence of gram-negative bacteria compared to gram-positive bacteria in the process of plaque buildup. Additionally, a mature plaque has more anaerobic bacteria because early colonizers consume oxygen, reducing the oxygen potential of the oral biofilm environment, which facilitates the proliferation of anaerobic bacteria [12]. Reichardt et al. [24] studied the influence of orthodontic appliances shortly (one week) after the start of the treatment. The findings revealed a qualitative increase in *Streptococcus mutans* at the premolar and molar regions, as identified by matrix-assisted laser desorption/ionization time-of-flight mass spectrometry.

*Pseudomonas* species, which are not typically present in the healthy oral microbiome, were found in orthodontic patients, as concluded by Sun et al. [25]. Some periodontal pathogens, including cysts of *Acanthamoeba* spp., were also identified [26]. Zibar Belasic et al. [27] observed a higher number of cariogenic bacteria than perio-pathogens in samples from patients treated with fixed appliance therapy. Gopalakrishnan et al. [28] noted a significant increase in sulfate-reducing bacteria in orthodontic patients compared to those not wearing any appliance. The rise in sulfate-reducing bacteria was attributed to the presence of metallic entities in the oral cavity, with colonization evident in the samples as black precipitates, which are bacterial by-products [28]. Merghni et al. [29] concluded that *Staphylococcus aureus*, due to their considerable adhesion to dental metal alloys (abiotic) and epithelial cells (biotic), were found in these samples, indicating biomaterial infections.

The study by Eckley et al. [30], consistent with the available literature, found an increase in *Porphyromonas gingivalis*, *Tannerella forsythia*, and *Treponema denticola* in orthodontic subjects. Additionally, dark-field microscopy revealed an increase in small and large spirochetes, non-motile rods, filaments, and fusiform and a decrease in coccoid forms and motile rods. These changes were reversed after the completion of treatment [30]. Marincak Vrankova et al. [7] suggested that the combined effect of periodontal bacteria and *Candida* species is more detrimental to periodontal health in the medium term after the insertion of orthodontic appliances than each of these microorganisms alone. There is also some evidence regarding detecting certain viruses in the periodontal deep pockets, indicating a superinfection associated with periodontal diseases, which needs further research [12].

Complications

Periodontal Complications: Gingivitis and Periodontitis

Arweiler et al. [10] confirmed that an imbalance in the oral cavity's ecosystem, due to either an excessive bacterial load or a compromised host immune system, can challenge both local and systemic health, leading to periodontal diseases and periodontitis. Gingivitis is a reversible, non-destructive inflammation of the gums characterized by accumulated plaque microflora at or near the gingival sulcus, which leads to local gingival irritation and signs of inflammation [31]. The reversible nature of this pathology suggests that it can be controlled at early stages. Most lesions are short-term; with close monitoring and proper plaque control methods, their progression can be hindered [32]. As the biofilm evolves and the number of pathogenic bacteria in the plaque continuously increases, so does the risk for periodontitis [33].

Periodontitis, a polymicrobial disease, results from an intensified inflammatory response to the benign microorganisms that have overgrown in subgingival spaces [9,12]. The transition in the subgingival microbiome marks the shift from healthy periodontium to gingivitis and eventually to periodontitis [34]. Periodontitis may follow a chronic course, causing irreversible clinical attachment loss [31] as evidenced by occult blood in saliva [35] and bone loss. It is important to note that even a small number of pathogenic bacteria are sufficient to damage periodontal tissues [12], and restoration of health can take time, even after resuming effective plaque control methods [36].

Other oral complications

Corrosion of Appliances

Microbial-induced corrosion represents a complex intersection of corrosion science and microbiology, where bacteria, fungi, and algae play pivotal roles. Among these, sulfate-reducing bacteria are particularly notorious. These bacteria are prevalent when orthodontic stainless steel appliances are used within the oral cavity, where they have been shown to cause corrosion. The literature extensively confirms that sulfate-reducing bacteria not only contribute to the corrosion of these appliances but are also among the causative agents of periodontal diseases. With an increased presence of sulfate-reducing bacteria in the biofilm of orthodontic patients, the risk of microbial-induced corrosion escalates [37]. Gopalakrishnan et al. [28] specifically investigated the corrosion of orthodontic metals by sulfate-reducing bacteria in patients treated with fixed appliances. Their study concluded that a rise in sulfate-reducing bacteria levels within the oral microflora correlates with increased corrosion of metallic appliances [28].

Caries/White Spot Lesions

As demonstrated by the studies mentioned above, orthodontic treatment can influence the proliferation of cariogenic bacteria, leading to the development of white spot lesions and dental caries. This effect is largely due to changes in the saliva's buffering capacity, which results in a more acidic environment. While stimulated salivary flow initially increases, it subsequently decreases to lower levels [35]. The resultant acidic pH fosters the growth of cariogenic bacteria such as *Streptococcus mutans* and *Lactobacillus*. This increase is often compounded by inefficient cleaning during orthodontic therapy, rendering the teeth more susceptible to decay.

Review

Literature Search Methodology

An electronic database search was conducted through PubMed, Google Scholar, and ScienceDirect to identify relevant studies. Initially, 98 articles were retrieved using the specified keywords. After reviewing the abstracts, articles that did not conform to the scope of this review or were written in languages other than English were excluded. To comprehensively demonstrate the microbial changes occurring in the periodontal environment due to orthodontic appliances, both recent and seminal studies, as well as earlier research findings, were included.

Microbiological Variables

Microbiological variables included the types of orthodontic appliances, surfaces, and treatment duration.

Fixed Appliance Therapy and Impact on Oral Microbiome

A study on Japanese patients that aimed to investigate microbial dynamics after fixed appliance therapy yielded significant findings. The research, conducted on 71 patients, involved collecting supragingival plaque and saliva samples at three stages: before the appliance was placed (T0), after six months of wear (T1), and after its removal (T2). A key finding was the significant increase in anaerobes and facultative anaerobes in both plaque and saliva, indicating dysbiosis in the periodontal environment. This dysbiosis was identified as a transitional stage from a healthy periodontium to periodontal disease [19].

Cantekin et al. [38] revealed that fixed appliance therapy negatively impacts oral health. The treatment heightened the risk of developing oral diseases due to plaque accumulation and microflora alteration. The effects were most critical in the first month after starting the treatment but diminished after debonding. Overall, the study concluded that fixed appliance therapy detrimentally affects patients' oral health [38,39]. Similarly, Eckley et al. [30] observed increased plaque index scores and probing depths in subjects undergoing fixed appliance therapy. On the other hand, a few studies found no significant difference in the periodontal status of adult patients who had undergone orthodontic treatment during adolescence [40]. No significant changes were observed in the periodontium post-therapy, and if any, they were not detrimental [40-42].

Yang et al. [43] conducted a study focusing specifically on the effects of fixed orthodontic appliances on the colonization of *Candida albicans* and *Streptococcus mutans*. These two microorganisms, typically synergistic, experienced altered colonization due to the long-term use of fixed appliances. This disturbance in the healthy balance between the fungus and bacterium was found to increase the risk of oral diseases [43].

Clear Aligner Therapy and Impact on Oral Microbiome

Clear aligner therapy is an excellent alternative to fixed appliance therapy for adolescents and, increasingly, for adults as well. The removable nature of these appliances facilitates better oral hygiene during orthodontic treatment, improving periodontal health and serving as a barrier against changes in microbial colonies. There remains a need to understand each phase of treatment (early, middle, late, and maintenance) to monitor changes in oral microflora and their progression over time [6]. Lucchese et al. [44] confirmed that removable appliances induce qualitative and quantitative changes in the bacterial population; however, these changes are transient and typically revert to optimal levels by the end of treatment.

Gong et al. [45] investigated gingival enlargement (GE) associated with orthodontic therapy, finding that the gingiva becomes swollen in reaction to microbial insults. The pathogenesis of GE induced by orthodontic treatment is multifactorial, with pathogens and inflammatory cytokines (interleukin-1 beta (IL-1β) and transforming growth factor beta 1 (TGF-β1)) identified as primary risk factors. Temporarily removing the appliance can benefit periodontal health by reducing pathogens and aiding GE recovery [45]. Existing literature has shown that clear aligner therapy has a lesser impact on the oral microbiome than fixed appliances do. Some studies have even noted a positive influence on gingival health, plaque index scores, and the occurrence of white spot lesions. The periodontal status did not deteriorate, and there was an improvement in pocket depths and gingival inflammation as treatment progressed. Nonetheless, it is crucial to note that proper plaque biofilm removal is essential, as the ridges, grooves, and abrasions on the aligners can harbor various microorganisms, leading to periodontal pathogenesis [6].

Lee et al. [46] studied the treatment of chronic periodontitis in three subjects who also exhibited maxillary anterior pathologic tooth migration using clear aligners. The study revealed improvements, with decreased probing depths, gingival recession, clinical attachment level, and tooth mobility during treatment [46]. Palone et al. [47] also noted better compliance and easier oral hygiene maintenance with clear aligners throughout treatment, resulting in reduced overall plaque buildup.

Hibino et al. [48] concluded that the insertion of removable appliances led to an increase in *Candida albicans* colonization. The study further highlighted the relationship between low salivary pH, removable appliances, and *Candida* species. It was observed that some non-*Candida* carriers transitioned to *Candida* carriers due to orthodontic treatment. However, the exact reason for this change remains unclear. The study emphasized that immunocompromised adolescents should be treated with caution to protect them against *Candida* infections [48].

Fixed Versus Removable Appliances

Clear aligners are becoming increasingly popular due to their cosmetic appeal, comfort, and ease of maintaining oral hygiene. However, they are not suitable for all cases. Complex orthodontic issues often require clinicians to opt for fixed appliance therapy. Both treatment options have their advantages and disadvantages. Lombardo et al. [49] compared the composition of subgingival microflora during the first six months of treatment with clear aligners and fixed appliances. The initial hypothesis was that there would be no difference between the two groups. Contrary to this, the results indicated that subgingival microflora increased in the fixed appliances group after three and six months, while it remained stable in the clear aligners group. The study concluded that the type of appliance significantly influences the microbial composition of subgingival microbiota [49].

A study by Perkowski et al. [26] found that during orthodontic treatment, the presence of *Enterococcus faecalis*, *E. faecium*, *Staphylococcus aureus*, *Escherichia coli*, and *Candida albicans* increased more significantly with fixed appliances than with removable appliances or no treatment at all. Jiang et al. [50] reported that clear aligners are preferable for enhancing periodontal health and reducing the risk of gingival inflammation, making them better suited for such patients. Rouzi et al. [6] noted that clear aligners produce controlled intermittent forces during treatment, which can be advantageous for rebuilding the periodontal membrane and improving periodontal health.

Wang et al. [51] found that the Invisalign system performs comparably to fixed appliance therapy in terms of treatment efficacy but highlighted the ease of oral hygiene maintenance with clear aligners, which might explain why clear aligners perform better in maintaining oral health.

Recent case reports have highlighted additional complications associated with fixed appliance therapy, particularly in relation to periodontal health. One such case involved a patient developing gingivitis on both upper and lower arches during treatment, underscoring the inflammatory response that can occur due to increased plaque accumulation and microbial shifts. Furthermore, mild recession of the upper incisors was observed, indicating that fixed appliances can contribute to periodontal tissue damage if not managed properly. Recent studies have also explored the use of mini implants (temporary anchorage devices (TADs)) in orthodontic treatments, highlighting their role in managing complex cases such as severe open bites. Mini implants are particularly effective for upper posterior intrusion, allowing for the precise movement of teeth without the need for external headgear. However, their use requires careful consideration of potential periodontal impacts. A case report documented mild recession of the upper incisors as a complication, emphasizing the need for vigilant periodontal monitoring and patient compliance to mitigate adverse effects. Additionally, this case revealed that patients undergoing fixed appliance therapy, particularly with mini implants, exhibited increased gingivitis and plaque accumulation, indicating the necessity for rigorous oral hygiene practices and regular periodontal assessments throughout the treatment duration [69].

Surfaces of the Appliances That Harbor Bacteria

Mini implants, while beneficial for anchorage and facilitating specific tooth movements, also present surfaces that can harbor bacteria, contributing to periodontal challenges. A case report indicated that the use of mini implants for upper posterior intrusion resulted in mild gingival recession, suggesting an increased risk of bacterial colonization around these devices. Furthermore, fixed appliances such as bands and brackets also contribute to plaque buildup and microbial shifts. The surfaces of orthodontic appliances can significantly influence periodontal health. For instance, a case report noted that the use of fixed appliances resulted in mild recession of the upper incisors, likely due to increased plaque retention and microbial colonization on appliance surfaces. This underscores the importance of selecting appropriate materials and designs that minimize plaque accumulation. Additionally, it highlights the necessity of vigilant oral hygiene practices to mitigate the adverse effects on periodontal tissues, particularly around bands and brackets where microbial buildup is most pronounced. This highlights the importance of maintaining impeccable oral hygiene and regular professional cleanings to prevent periodontal complications associated with the use of mini implants and other orthodontic components. The report emphasized that surfaces of elastomeric ligatures and bands, in particular, tend to accumulate more plaque, leading to increased inflammation and bleeding, further complicating periodontal health [69].

Türkkahraman et al. [52] conducted a split-mouth study on 21 subjects to compare elastomeric/O rings and steel ligature techniques. A significant finding was that elastomeric ligatures are more prone to plaque accumulation in fixed appliance therapy than steel ligatures, providing crucial insight for orthodontic professionals. Furthermore, signs of inflammation, such as bleeding, were observed at the sites with elastomeric ligatures, highlighting the need for further research to fully understand the implications [52]. Another study by Palone et al. [47] also supported the use of steel ligature over conventional (elastomeric) and self-ligating ligatures, emphasizing the necessity for additional research in this area.

Alves de Souza et al. [53] reported that organisms such as *Tannerella forsythia* and *Prevotella nigrescens* were found in significantly higher numbers at the elastomeric ligatures, while *Porphyromonas gingivalis*, *Actinobacillus actinomycetemcomitans*, and *Prevotella intermedia* showed no difference. Lombardo et al. [54] explored the differences between labial and lingual orthodontic appliances, observing noticeable differences in lingual appliances 4-8 weeks after bonding. After eight weeks, increased gingival inflammation and a rise in *Streptococcus mutans* counts were noted, although *Lactobacillus* counts, salivary buffer capacity, and salivary flow rate remained consistent between the two groups.

Chen et al. [18] studied microbial changes around orthodontic brackets and bands, finding that bands led to more plaque buildup and microbial shifts than brackets, with 12 novel bacterial species identified. Kim et al. [55] reported a higher prevalence of pathogenic microflora, bleeding on probing, and deeper pockets around orthodontic bands, underlining the potential risks associated with these appliances. Mini-screw implants (MSIs) are commonly used in orthodontics for anchorage [55].

Mishra et al. [56] researched microbial colonization around MSIs and concluded that bacterial buildup occurs within 24 hours, a crucial finding for understanding the timeline of bacterial colonization. The peri-mini-implant crevicular fluid contained higher numbers of staphylococci, anaerobic cocci, and facultative enteric commensal bacteria compared to gingival crevicular fluid, necessitating careful monitoring. Moreover, failed mini-screw implants showed higher proportions of staphylococci, *Enterobacter *spp., and *Parvimonas micra*, factors linked to the stability and durability of the implant [56].

Contaldo et al. [13] noted that some orthodontic components, such as bonded molar brackets, ceramic brackets, and elastomeric ligatures, increase the risk of periodontal diseases and caries, stressing the importance of careful material selection. Santonocito et al. [57] emphasized that the current gingival status of the patient, optimum oral hygiene maintenance, and susceptibility to caries are critical factors when selecting an appliance for an orthodontic patient. They recommend avoiding ceramic brackets and elastomeric ligatures for patients with a thin gingival biotype, poor oral hygiene, or high caries risk, as these sites are more prone to microbial colonization and the progression of periodontal diseases [57].

Role of the Duration of Appliance Wear and Timing of Treatment

The timing and duration of orthodontic treatment play vital roles in microbial alteration. Treatment with fixed appliances tends to take longer than with removable appliances. However, the overall impact on oral health depends on various factors, including the complexity of the case and the patient's oral hygiene practices. In some instances, removable appliances used over a long period, especially in cases of poor oral hygiene, can result in worse periodontal outcomes compared to fixed appliances. Therefore, it is essential to emphasize the importance of maintaining good oral hygiene regardless of the type of appliance used. As demonstrated by Lucchese et al. [58], the duration the appliance is used in the mouth is a significant variable to consider. The treatment duration is shorter with removable appliances, and they can also be easily removed, allowing for better oral hygiene maintenance due to their less plaque-retentive surfaces compared to fixed appliances. They pointed out that all types of orthodontic treatment can pose some challenges to the periodontium; however, the impact of fixed appliance therapy, with its longer duration and the notable difficulty in maintaining oral hygiene, must be carefully considered [58]. Santonocito et al. [57] confirmed that minimizing the duration of use for fixed appliances is important to prevent excessive microbial buildup.

Koopman et al. [59] studied the variables impacting changes in the oral environment after orthodontic treatment. They found more pronounced changes related to patients' current gingival health, the type of orthodontic treatment, and the timing of the procedure [59]. Few studies have been conducted to explore the impact of the duration, the start and end of orthodontic treatment, and how each stage differs from the others. The microbiological changes observed before, during, and after treatment show how each phase functions and determine the measures necessary to minimize the consequences of microbial shifts.

Initial stage: The existing literature indicates that microbial changes become noticeable shortly (one or two weeks) after the initiation of treatment with either fixed or removable appliances. However, if treatment is discontinued, microbial levels tend to return to pre-treatment levels within 30 days of appliance removal [24,44,47]. This is attributed to the new surfaces, such as metal brackets, bands, and wires, which support biofilm buildup, leading to mild gingivitis in the early stages of treatment. However, some theories suggest that the progression and maturation of the oral microbiome continue until 90 days after treatment.

Mid-term stage: On the other hand, long-term observations for at least six months after the removal of orthodontic appliances indicated that the levels of *Tannerella forsythia* and *Fusobacterium nucleatum* initially increased but returned to normal levels after a few months [60,61]. However, Kim et al. [55] found that the levels of *Tannerella forsythia* remained elevated during the first six months without any decrease, suggesting a need for further research involving extended durations to draw accurate conclusions. Additionally, Koopman et al. [59] observed that populations of periodontal pathogens such as *Selenomonas* and *Porphyromonas* increased during orthodontic treatment. If oral hygiene is not adequately maintained, late-stage gingivitis (early periodontitis) becomes clinically visible as teeth begin to move, resulting in deeper pockets. During this stage, certain bacteria become more prevalent than others.

Late stage: Thornberg et al. [61] concluded that the number of periodontal pathogens increased during the first six months of orthodontic treatment but then decreased and returned to pre-treatment levels over the course of 12 months. In contrast, another study reported that bacteria such as *Streptococcus*, *Rothia*, and *Haemophilus* were abundant toward the end of or after the completion of orthodontic treatment [59]. For these reasons, it is crucial to adhere strictly to oral hygiene protocols. The oral environment should also reach equilibrium once the teeth have achieved their desired position and alignment. Any further disturbances to oral microbiology may lead to chronic periodontal issues.

Post-treatment: Once the appliance is removed, controlling plaque buildup becomes easier, and cleaning vulnerable surfaces is more convenient. While the periodontal changes are largely reversible once a healthy oral microbiome balance is reestablished, the extent of reversibility is greatly dependent on individual patient factors. Some researchers have found that the periodontal changes induced by orthodontic treatment were only partially reversible, even three months after the removal of the appliance [59,62].

Table 1 presents a summary of microbial changes after orthodontic treatment.

**Table 1:** Summary of Microbial Changes After Orthodontic Treatment

Stage	Microbial changes	Bacteria types
Initial stage	Microbial changes become noticeable shortly (one or two weeks) after treatment initiation. Mild gingivitis may occur. Microbial levels tend to return to pre-treatment levels within 30 days after appliance removal.	Metal brackets, bands, and wires support biofilm buildup
Mid-term stage	Levels of *Tannerella forsythia* and *Fusobacterium nucleatum* initially increased but returned to normal levels after a few months. Populations of periodontal pathogens such as *Selenomonas* and *Porphyromonas* increased. Without adequate oral hygiene, late-stage gingivitis (early periodontitis) may become visible.	*Tannerella forsythia*, *Fusobacterium nucleatum*, *Selenomonas*, *Porphyromonas*
Late stage	The number of periodontal pathogens increased during the first six months but then decreased to pre-treatment levels over 12 months. Some bacteria such as *Streptococcus*, *Rothia*, and *Haemophilus* were abundant toward the end or after treatment completion.	Periodontal pathogens, *Streptococcus*, *Rothia*, *Haemophilus*
Post-treatment	Controlling plaque buildup becomes easier. Periodontal changes are largely reversible once a healthy oral microbiome balance is reestablished. However, some changes may only be partially reversible, even three months after appliance removal.	General oral microbiome balance

Effective Oral Hygiene Practices During Orthodontic Treatment

Effective oral hygiene practices are crucial during orthodontic treatment to prevent periodontal complications. The inclusion of mini implants in orthodontic treatment protocols necessitates enhanced oral hygiene practices to prevent periodontal issues. Effective plaque control around mini implants is critical, as these devices can serve as niches for bacterial accumulation. A case report emphasized the importance of follow-up visits and long-term care, revealing that patients might require additional periodontal treatments, such as gingival grafts, to achieve stable outcomes post-treatment. This underscores the importance of patient compliance with oral hygiene routines and regular dental visits to monitor and manage the health of periodontal tissues surrounding mini implants, ensuring successful treatment outcomes and minimizing complications such as gingival recession. Additionally, follow-up visits and long-term care are essential to ensure the stability of orthodontic treatment outcomes. The need for potential gingival grafts post-treatment highlights the importance of continuous periodontal monitoring and tailored oral hygiene protocols, including the use of specialized orthodontic brushes, interdental cleaning tools, and possibly antiseptic mouthwashes, to ensure plaque and bacterial accumulation are effectively controlled throughout and after the treatment process [69].

Maintaining good oral hygiene and plaque control regimens during orthodontic treatment is essential [63]. It is crucial for patients to understand the importance of maintaining good oral hygiene throughout their treatment and to make regular follow-up visits to the dentist to mitigate the risk of plaque buildup and the growth of periodontopathogens and cariogenic bacteria. These issues can develop soon after treatment begins, as these alterations occur shortly after the initiation of treatment [57]. Soni et al. [64] conducted a 90-day randomized, single-blind study to compare the effectiveness of different methods for controlling plaque in patients undergoing fixed appliance therapy. They found that orthodontic toothbrushes and water jets successfully controlled plaque and promoted oral hygiene [64]. Özdemir et al. [65] studied the impact of brushing with Cervitec gel once a day to reduce plaque accumulation around teeth and fixed appliances. While the results did not significantly affect subgingival dental plaque levels, an improvement was observed on day 14, as indicated by a decrease in the Quigley-Hein Plaque Index score. Additionally, after receiving professional prophylaxis and oral hygiene instructions, a positive outcome and noteworthy improvement were observed clinically and pathologically [65].

Bauer Faria et al. [66] reported that *Zingiber officinale* mouthwash effectively controlled dental biofilm and reduced gingival inflammation, although they suggested that its flavor needs to be improved. Vidović et al. [67] concluded that using an octenidine (OCT)-based antiseptic reduced the risk of gingivitis and the number of subgingival bacteria in the first three months of fixed orthodontic treatment. Zibar Belasic et al. [27] found that *Streptococcus mutans* were better controlled with fluorides than chlorhexidine, and *Streptococcus sobrinus* and *Aggregatibacter actinomycetemcomitans* responded well to chlorhexidine. By introducing different methods to control the risk factors for periodontal diseases and consulting with the dentist, an overall successful orthodontic treatment outcome can be expected. Nonetheless, the gold standard strategy to prevent the risk of periodontal disease is mechanical removal by regular tooth brushing (using orthodontic brushes and flossing) [10].

Discussion

This narrative review extensively examined the literature and found a consensus regarding the negative impact of orthodontic therapy on the periodontal environment. Studies by Kado et al. [19] and Cantekin et al. [38] concluded that the use of fixed appliances led to a transition from a healthy periodontium to a diseased state, with some recovery observed after debonding. The period immediately after appliance placement was identified as crucial for maintaining oral hygiene to prevent adverse changes [19,38].

Conversely, Koopman et al. [59] and Pan et al. [62] noted only partial recovery of periodontal health months after appliance removal, emphasizing the importance of patient compliance with oral hygiene protocols. The literature generally supports clear aligners over fixed appliances due to their ease of removal, which facilitates better oral hygiene and reduces the risk of periodontal disease [6,44,47]. Significant findings by Yang et al. [43] and Hibino et al. [48] indicated an increase in *Candida albicans* during orthodontic treatment, highlighting the challenges posed by low pH and microbial dynamics, which may lead to higher risks of *Candida*-related diseases.

Contrary perspectives were highlighted by Sadowsky et al. [40], who found minimal impact of orthodontic appliances on long-term periodontal health. Their study compared individuals treated orthodontically with a control group and noted no significant differences in periodontal diseases, although some areas exhibited slightly higher risks of mild to moderate periodontal issues [40]. Gomes et al. [41] also reported no negative impacts from orthodontic treatment on periodontal pocketing depth and clinical attachment loss, suggesting that periodontal diseases could develop independently of orthodontic treatment factors. Bollen et al. [68] conducted a systematic review that reported inconsistent effects on gingivitis and clinical attachment loss, indicating that orthodontic appliances pose little risk to the periodontium. Chen et al. [18] highlighted the increased richness of the subgingival microbiome following orthodontic therapy, emphasizing the need to understand these microbial shifts.

Overall, while there is substantial evidence of the effects of orthodontic treatment on the periodontal environment, further research is necessary to fill the gaps in understanding how these treatments impact microbial changes, especially concerning the potential involvement of other microorganisms such as viruses and protozoa. Additionally, examining the effects in immunocompromised patients and assessing the systemic health impacts of microbial shifts are crucial areas for future investigation. The role of gram-negative bacteria in periodontal disease development, particularly following orthodontic treatment, warrants continued attention [12,13,22,23].

## Conclusions

This review highlights certain findings. First, periodontopathogens, cariogenic bacteria, and corrosion-inducing bacteria increase during orthodontic treatment, which subsequently increases the risk of gingivitis, periodontitis, dental caries, and appliance corrosion. Second, fixed appliance therapy poses a greater risk of periodontal issues compared to clear aligners due to their longer duration and continuous wear. On that note, strict plaque control is crucial during the early and mid-treatment stages to ensure that the oral cavity returns to a healthy state in the late and post-treatment stages. Surfaces such as ceramic brackets, molar bands, mini-implant screws, and elastomeric ligatures are particularly prone to plaque accumulation. Effective plaque control and improved treatment outcomes were achieved using orthodontic toothbrushes, mouthwashes, and professional prophylaxis.
